# Supplementary material for: Clinical and laboratory characteristics of adolescents and young adults with sickle cell disease at steady state in Uganda
Source: PLOS Glob Public Health. 2026 Mar 30;6(3):e0005898. doi: 10.1371/journal.pgph.0005898 (PMC13035150; doi:10.1371/journal.pgph.0005898)
Supplement: Attachment — Submitted filename: RESPONSE TO REVIEWERS.docx [file pgph.0005898.s002.docx]

**22/02/2026**

**RESPONSE TO REVIEWERS**

**Manuscript ID:** PGPH-D-26-00114
**Title:** Clinical and Laboratory Characteristics of Adolescents and Young Adults with Sickle Cell Disease at Steady State in Uganda

We sincerely thank the reviewers for their careful evaluation of our manuscript and for the constructive feedback. We are encouraged by their positive assessment of the study’s methodological rigor, clarity, and relevance. We have addressed all comments as detailed below.

**Reviewer #1**

**Comment**

The study presents original research and is a valuable continuation of work at Mulago Hospital. The conclusion, however, does not reflect important findings related to nutrition and disease-modifying therapy.

**Response**

We agree and have revised the conclusion to incorporate the high proportion of undernutrition and the widespread uptake of hydroxyurea, emphasizing the need for integrated steady-state care.

**Revision made**

The conclusion now reads:

*Adolescents and young adults with SCD at steady state exhibit persistent hematologic and biochemical abnormalities, altered hemodynamics, and a high proportion of undernutrition. High hydroxyurea uptake reflects expanding access to disease-modifying therapy. These findings not only provide a baseline reference but also support integrated steady-state care that combines routine monitoring, nutritional assessment, sustained treatment delivery, and future research in Uganda.*

**Comment**

Please review the sentence in lines 39–40 of the abstract.

**Response**

We corrected the grammatical error.

**Revision made**

*However, data describing the clinical and laboratory characteristics of adolescents and young adults with SCD at steady state in sub-Saharan Africa are limited.*

**Reviewer #2**

**Comment 1**

Please correct the second sentence in the abstract.

**Response**

This sentence has been corrected as described above.

**Comment 2**

Please provide a reference or describe the criteria used for determining underweight participants.

**Response**

We clarified nutritional classification using World Health Organization age-appropriate criteria for adolescents and adults.

**Revision made (Methods section)**

*Body mass index (BMI) was calculated as weight in kilograms divided by height in meters squared. Nutritional status was classified using World Health Organization criteria: BMI-for-age Z-score < −2 standard deviations for adolescents aged 13–18 years, and BMI <18.5 kg/m² for participants aged ≥19 years.*

**Comment 3**

Please include the mean and standard deviation for participant weights in Table 1.

**Response**

We added mean ± standard deviation body weight to Table 1 and reported this value in the Results section. We have also further stratified the proportion of participants with underweight based on age (adolescents and young adults).

**Revisions made**

**Table 1 now includes:**

*Weight, kg (mean ± SD): 46.5 ± 10.1*

**Results section now states:**

*The mean body weight of participants was 46.5 ± 10.1 kg.*

**Additional clarification added to Results**

Based on World Health Organization age-appropriate criteria, 32 participants (53.3%) were underweight. Underweight prevalence was higher among adolescents (56.5%) compared with young adults aged ≥19 years.

Thank you,

Abdul Magala Ssekandi, MBChB, MSc.
